# ^161^Tb Radioimmunotherapy as a Treatment for CD30-Positive Lymphomas

**DOI:** 10.2967/jnumed.124.268805

**Published:** 2025-06

**Authors:** Elisa Rioja-Blanco, Yara Banz, Christoph Schlapbach, Urban Novak, Tanja Chiorazzo, Nicole L. Bertschi, Peter Bernhardt, Pascal V. Grundler, Nicholas P. van der Meulen, Michal Grzmil, Roger Schibli, Martin Behe

**Affiliations:** 1 Center for Radiopharmaceutical Sciences, PSI Center for Life Sciences, Villigen-PSI, Switzerland;; 2 Department of Chemistry and Applied Biosciences, Institute of Pharmaceutical Sciences, ETH Zurich, Zurich, Switzerland;; 3 Institute of Tissue Medicine and Pathology, University of Bern, Bern, Switzerland;; 4 Department of Dermatology, Inselspital, Bern University Hospital, University of Bern, Bern, Switzerland;; 5 Department of Medical Oncology, Inselspital, Bern University Hospital, University of Bern, Bern, Switzerland;; 6 Department of Medical Radiation Sciences, Institute of Clinical Sciences, Sahlgrenska Academy at University of Gothenburg, Gothenburg, Sweden;; 7 Department of Medical Physics and Biomedical Engineering, Sahlgrenska University Hospital, Gothenburg, Sweden; and; 8 Laboratory of Radiochemistry, PSI Center for Nuclear Engineering and Sciences, Villigen-PSI, Switzerland

**Keywords:** lymphoma, CD30, radioimmunotherapy, ^161^Tb, phosphoproteomics

## Abstract

Lymphoma remains a significant health concern, necessitating innovative treatment approaches. ^161^Tb’s coemission of ultra-short-range conversion and Auger electrons, with its medium-energy β^−^-particles, may enable the elimination of single cells and small clusters present in circulation, improving therapeutic outcomes. In this study, we compared ^161^Tb radioimmunotherapy targeting CD30—a receptor overexpressed in lymphomas—with ^177^Lu-radiolabeled therapy to evaluate the effect of ^161^Tb’s additional emission of conversion and Auger electrons. **Methods:** The ability of ^161^Tb- and ^177^Lu-radiolabeled anti-CD30 antibody (cAC10) to reduce cell viability and survival and induce DNA damage was evaluated in vitro in CD30-positive T-cell lymphoma cell lines. The biodistribution, dosimetry, and therapeutic effect of both radioimmunoconjugates were studied in a xenograft mouse model. Xenografts treated with [^161^Tb]Tb-cAC10 and [^177^Lu]Lu-cAC10 were submitted for quantitative proteomics and phosphoproteomics analyses, followed by bioinformatics analysis. **Results:** [^161^Tb]Tb-cAC10 demonstrated superior and CD30-specific cytotoxicity across a panel of T-cell lymphoma cell lines. In vivo studies showed a favorable biodistribution, with high tumor uptake (31.0 ± 7.4 percentage of injected activity per mass of tissue after 48 h) and a higher tumor-absorbed dose for ^161^Tb. Importantly, a single administration of the ^161^Tb-radiolabeled compound significantly prolonged survival time compared with an equal injected activity of [^177^Lu]Lu-cAC10 (median survival, 41 d vs. 21 d). Phosphoproteomic analysis revealed that both [^177^Lu]Lu-cAC10 and [^161^Tb]Tb-cAC10 induced alterations in pathways regulating DNA damage response and cell cycle, among others, with ^161^Tb inducing more pronounced changes than did ^177^Lu. **Conclusion:** ^161^Tb radioimmunotherapy was an effective therapy for CD30-positive T-cell lymphomas. To our knowledge, this is the first evaluation of this therapeutic radionuclide for the treatment of hematologic malignancies. Additionally, in vivo phosphoproteomics provided insights into the biologic processes and pathways regulated by radioimmunotherapy administration, further supporting the superior efficacy of ^161^Tb over ^177^Lu.

## Visual Abstract

**Figure:**
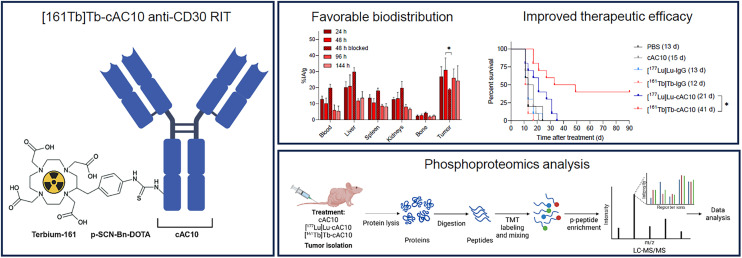

Lymphomas affect over 500,000 new patients worldwide annually, leading to more than 200,000 deaths each year (1). Among malignant lymphomas, T-cell lymphomas represent a relatively rare and diverse subgroup, comprising over 20 distinct entities. Their management is challenging because of the heterogeneity of these diseases, ranging from indolent to aggressive forms (2). T-cell lymphomas are neglected clinical entities with limited therapeutic options, primarily consisting of multiagent chemotherapy and stem cell transplantation. Additional options include targeted therapy, radiotherapy, and immunotherapy. The anti-CD30 antibody–drug conjugate brentuximab vedotin represents 1 of the few targeted therapeutic options available (3–5). However, its clinical benefits are limited. In T-cell lymphomas, the median response duration to monotherapy is only 7.6 mo (6). Moreover, cumulative peripheral neuropathy constitutes a severe side effect, limiting prolonged use of the drug (7). Despite the initial effectiveness of these therapies, advanced and relapsed lymphomas remain challenging, particularly aggressive types with relapse rates as high as 60%, highlighting the need for novel therapeutic strategies (8,9).

Radioimmunotherapy with Auger-electron emitters is a promising alternative to overcome these treatment limitations. Radioimmunotherapy was introduced into clinical practice over 2 decades ago with the approval of [^90^Y]Y-ibritumomab tiuxetan and ^131^I-tositumomab, anti-CD20 antibodies coupled with the β^−^-particle emitters ^90^Y (β^−^-particles of an average energy [E_β−average_], 934 keV; half-life [*t*_1/2_], 2.7 d; maximum tissue range, 11 mm) and ^131^I (E_β−average_, 187 keV; *t*_1/2_, 8 d; maximum tissue range, 2.4 mm), respectively (10,11). Despite demonstrating clinical efficacy and safety, ^131^I-tositumomab was discontinued and [^90^Y]Y-ibritumomab tiuxetan is rarely used (12). Other than pharmacologic issues with the in vivo deiodination of ^131^I-tositumomab (13), the high energy of the β^−^-particles emitted by ^90^Y is ineffective in the killing of small tumor cell clusters and single cancer cells (14). In recent years, the short-range β^−^-emitter ^177^Lu (E_β−average_, 134 keV; energy of the photon [E_γ_], 113 and 208 keV; *t*_1/2_, 6.65 d; maximum tissue range, ∼2 mm) has become the most widely used therapeutic radionuclide (15), as exemplified by the clinical and commercial success of ^177^Lu-PSMA-617, approved for the treatment of castration-resistant prostate cancer, and ^177^Lu-DOTATATE, used in the treatment of neuroendocrine tumors.

More recently, ^161^Tb has gained attention as a potentially superior therapeutic radionuclide over ^177^Lu. Similar to ^177^Lu,^ 161^Tb decays by emission of β^−^-particles (E_β−average_, 154 keV; E_γ_, 49 and 75 keV; *t*_1/2_, 6.95 d), (16); however, ^161^Tb coemits a substantial number of conversion and Auger electrons, which display an ultrashort range in tissue (<5 μm). Due to their high linear energy transfer (4–26 keV/μm), these conversion and Auger electrons are especially effective in eliminating single cells and small cell clusters, such as those present in circulation and disseminated stages of lymphoma (14,17). However, to the best of our knowledge, the therapeutic efficacy of ^161^Tb has not been explored in hematologic cancers.

This study compared radioimmunotherapy with ^161^Tb for the treatment of T-cell lymphomas with the current benchmark ^177^Lu to assess the contribution of ^161^Tb’s coemission of conversion and Auger electrons. CD30 served as the clinically validated target, as it is highly overexpressed in many lymphoma subtypes, including T-cell lymphomas, but minimally expressed in healthy tissue (18,19). CD30 targeting was achieved by coupling the cell-internalizing, full-length, chimeric antibody anti-CD30 antibody (cAC10) to the chelator DOTA, enabling radionuclide labeling. To our knowledge, this is the first preclinical evaluation of ^161^Tb for the treatment of a hematologic malignancy. In vivo proteomics and phosphoproteomics analyses of lymphoma tumor cell responses to radioimmunotherapy were also conducted.

## MATERIALS AND METHODS

A detailed explanation of all experimental procedures is provided in the supplemental materials (supplemental materials are available at http://jnm.snmjournals.org) (20–25).

### Antibody Conjugation, Radiolabeling, and Quality Control

cAC10 antibody (IgG1 κ) was produced by Proteogenix. Conjugation of the antibody to DOTA was performed by incubation with p-SCN-Bn-DOTA (Macrocyclics). No-carrier-added ^177^Lu was purchased from ITM Medical Isotopes GmbH. No-carrier-added ^161^Tb was produced at Paul Scherrer Institute (26).

### Cell Culture and Cell Assays

The CD30-positive T-cell lymphoma cell lines Karpas 299, Mac2A, and Myla were provided by Christoph Schlapbach (Inselspital). Jurkat cells were used as a negative control. Cell lines were cultured in standard conditions.

### In Vivo Experiments

Four-week-old female athymic nude mice (Crl:NU(NCr)-*Foxn1^nu^*; Charles River Laboratories) were used in all studies in compliance with Swiss Animal Welfare regulations. The biodistribution, dosimetry, and therapeutic effect of the radioimmunoconjugates were assessed in a subcutaneous Karpas 299 xenograft model. Hematologic toxicity after treatment was evaluated in non–tumor-bearing mice.

## RESULTS

### Synthesis and Quality Control

The radiometal chelator DOTA was coupled to the cAC10 antibody at a ratio of 3.1 chelators per antibody. DOTA-functionalized [^177^Lu]Lu-cAC10 and [^161^Tb]Tb-cAC10 were obtained at high specific activities (≥0.3 MBq/μg) and radiochemical purities (>90%) (Supplemental Fig. 1). Both radioimmunoconjugates showed high plasma stability, with at least 90% of the radioactivity coupled to the immunoconjugates after 7 d (Supplemental Fig. 2). The binding affinity was in the lower nanomolar range (Supplemental Fig. 3), consistent with a previously published findings (27).

### Cytotoxicity

**FIGURE 1.**
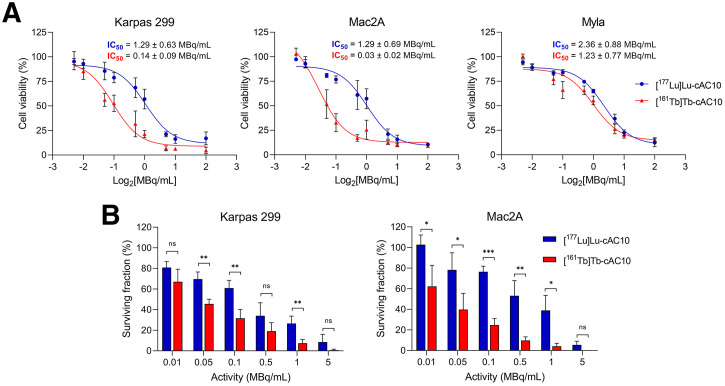
In vitro cytotoxic effects of [^161^Tb]Tb-cAC10 vs. [^177^Lu]Lu-cAC10 in CD30-positive lymphoma cell lines. (A) Viability of the CD30-positive lymphoma cell lines Karpas 299, Mac2A, and Myla after treatment with different activity concentrations of [^177^Lu]Lu- or [^161^Tb]Tb-cAC10 (0–20 MBq/mL) (mean ± SD; *n* = 3). (B) Cell survival (clonogenic assay) of Karpas 299 and Mac2A cell lines after treatment with increasing activity concentrations of ^177^Lu- and ^161^Tb-radiolabeled radioimmunoconjugates (0–5 MBq/mL) (mean ± SD; *n* = 3). *P ≤ 0.05; **P ≤ 0.01; ns = nonsignificant.

The cytotoxic effects of [^161^Tb]Tb-cAC10 and [^177^Lu]Lu-cAC10 were compared in 3 CD30-positive T-cell lymphoma cell lines (Karpas 299, Mac2A, and Myla). In all tested cell lines, [^161^Tb]Tb-cAC10 was more potent in reducing cell viability compared with the ^177^Lu-radiolabeled counterpart, as reflected in the calculated half-maximal inhibitory concentration (IC_50_) values for Karpas 299 (0.14 ± 0.09 and 1.29 ± 0.63 MBq/mL, respectively), Mac2A (0.03 ± 0.02 and 1.29 ± 0.69 MBq/mL, respectively), and Myla (1.23 ± 0.77 and 2.36 ± 0.88 MBq/mL, respectively) (Fig. 1A). The largest difference was observed in the Mac2A cell line, where [^161^Tb]Tb-cAC10 was 43-fold more potent than [^177^Lu]Lu-cAC10. For Karpas 299 and Myla cell lines, the IC_50_ values for ^161^Tb were 9.2- and 1.9-fold lower than those for ^177^Lu, respectively. Importantly, these differences in cytotoxicity were not due to differences in cell uptake or subcellular localization between these radiolanthanides (Supplemental Figs. 4 and 5). The cellular uptake and cytotoxicity of both radioimmunoconjugates correlated with the differing CD30 expression among the 3 cell lines (Supplemental Figs. 6 and 7). In addition, the potent cytotoxicity was CD30-dependent, as demonstrated by 2 independent control experiments (Supplemental Fig. 8). [^161^Tb]Tb-cAC10 and [^177^Lu]Lu-cAC10 were tested in the CD30-negative Jurkat cell line (Supplemental Fig. 8A), whereas a radiolabeled control antibody (IgG) was tested in Karpas 299 cells (Supplemental Fig. 8B). The calculated IC_50_ values were 249- and 760-fold higher for ^161^Tb and 19- and 32-fold higher for ^177^Lu, respectively, when compared with the most sensitive cell line, Mac2A.

Colony-forming assays demonstrated that the ^161^Tb-radiolabeled antibody was also more effective in reducing cell survival compared with the ^177^Lu radioimmunoconjugate in the Karpas 299 and Mac2A cell lines (Fig. 1B). The most pronounced difference between both radioimmunoconjugates was observed in the Mac2A cell line, where less than 10% of the cells treated with 0.5 MBq/mL of [^161^Tb]Tb-cAC10 survived, whereas a 10-fold higher activity concentration of [^177^Lu]Lu-cAC10 was needed to obtain a similar effect.

### DNA Damage

**FIGURE 2.**
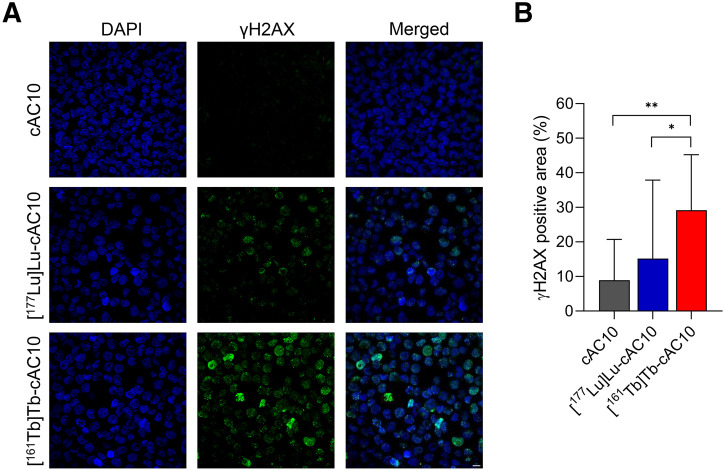
DNA damage assessment (γH2AX) in Karpas 299 lymphoma cells induced by [^161^Tb]Tb-cAC10 vs. [^177^Lu]Lu-cAC10. (A) Representative DAPI (blue) and γH2AX (green) stainings in control (cAC10) and radioimmunoconjugate-treated ([^177^Lu]Lu- or [^161^Tb]Tb-cAC10) cells. Scale bar = 10 μm. (B) Quantification of γH2AX-positive stained area in control and radioimmunoconjugate-treated cells, expressed as percentage of the area normalized by DAPI area (mean ± SD; *n* = 3). *P ≤ 0.05; **P ≤ 0.01.

γH2AX immunocytochemistry showed a 1.9-fold increase in the percentage of DNA double-strand break in Karpas 299 cells treated with the ^161^Tb-radiolabeled compound compared with its ^177^Lu-radiolabeled counterpart (Figs. 2A and 2B). These findings confirm ^161^Tb’s higher double-strand break induction.

### Biodistribution

**FIGURE 3.**
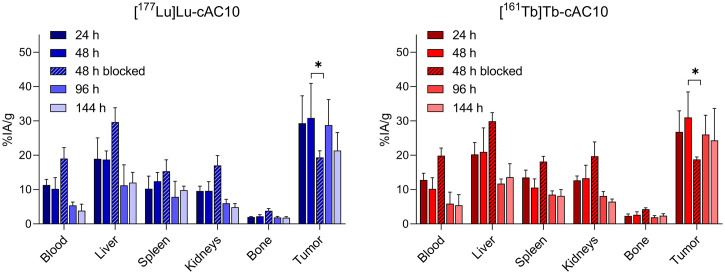
Biodistribution was similar among Karpas 299 tumor–bearing mice intravenously injected with 1 MBq (30 μg) of [^177^Lu]Lu-cAC10 or [^161^Tb]Tb-cAC10 at 24, 48, 96, and 144 h, expressed as %IA/g (mean ± SD; *n* = 5). *P ≤ 0.05.

[^177^Lu]Lu-cAC10 and [^161^Tb]Tb-cAC10 biodistribution in Karpas 299-derived xenografts was assessed in a time-dependent study. Both compounds showed a favorable and similar biodistribution, with high tumor uptake (up to 30 percentage of injected activity per mass of tissue [%IA/g] after 48 h). Tumors exhibited the highest compound accumulation at all time points (Fig. 3; Supplemental Table 1). Liver and spleen uptake was approximately 20 and 12 %IA/g, respectively, after 48 h, decreasing at 144 h. Tumor accumulation was CD30-dependent, as demonstrated by the significant reduction in tumor uptake in the blocking condition (Fig. 3; Supplemental Table 1). Dosimetry calculations revealed that the mean absorbed dose of [^161^Tb]Tb-cAC10 in all organs, including tumors, was 1.6-fold higher than that of the lutetium-radiolabeled counterpart (Supplemental Table 2).

### Therapy/Radioimmunotherapy Study

**FIGURE 4.**
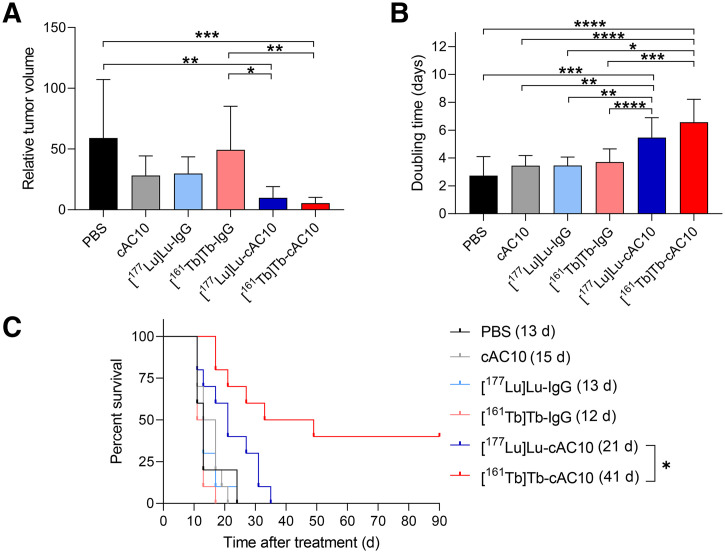
Comparison of the therapeutic effect of [^161^Tb]Tb-cAC10 and [^177^Lu]Lu-cAC10 in Karpas 299 tumor–bearing mice. Relative tumor volume (A) and tumor doubling time (B) among 6 study groups at day 11 (time at which first mouse reached endpoint) (mean ± SD; *n* = 10). *P ≤ 0.05; **P ≤ 0.01; ***P ≤ 0.001; ****P ≤ 0.0001 (1-way ANOVA, Tukey multiple comparison test). (C) Kaplan–Meier plot of control (phosphate-buffered solution [PBS], cAC10, [^177^Lu]Lu-IgG, and [^161^Tb]Tb-IgG) and treatment groups ([^177^Lu]Lu-cAC10 and [^161^Tb]Tb-cAC10) (*n* = 10). *P ≤ 0.05.

The therapeutic efficacy of [^177^Lu]Lu-cAC10 and [^161^Tb]Tb-cAC10 radioimmunoconjugates was evaluated in vivo after the administration of 2 MBq of either radiolabeled compound. The injected activity was selected based on previous study results (Supplemental Fig. 9) to enable the direct comparison of both radionuclides and evaluate the potential contribution of ^161^Tb ‘s additional coemission of conversion and Auger electrons. Control mice (phosphate-buffered solution, cAC10, [^177^Lu]Lu-IgG, and [^161^Tb]Tb-IgG) demonstrated rapid tumor growth, leading to euthanasia between days 11 and 24 in all cases (Supplemental Fig. 10). Eleven days after the start of the treatment, mice in the control groups had significantly higher relative tumor volumes and lower tumor-doubling times compared with both treatment groups (Figs. 4A and 4B). No significant differences were observed in median survival times between control groups (Fig. 4C). Treatment with a single dose of 2 MBq [^177^Lu]Lu-cAC10 led to significant arrest of tumor growth (Figs. 4A and 4B) and consequent survival prolongation (Fig. 4C). However, all tumors eventually eluded treatment, and no mice survived by the end of the experiment. In contrast, mice treated with 2 MBq of [^161^Tb]Tb-cAC10 had a significantly prolonged median survival time compared with those in the ^177^Lu-treated group (41 d vs. 21 d) (Fig. 4C). Importantly, treatment with [^161^Tb]Tb-cAC10 led to complete tumor elimination in 4 mice, who remained tumor-free until the end of the experiment.

**FIGURE 5.**
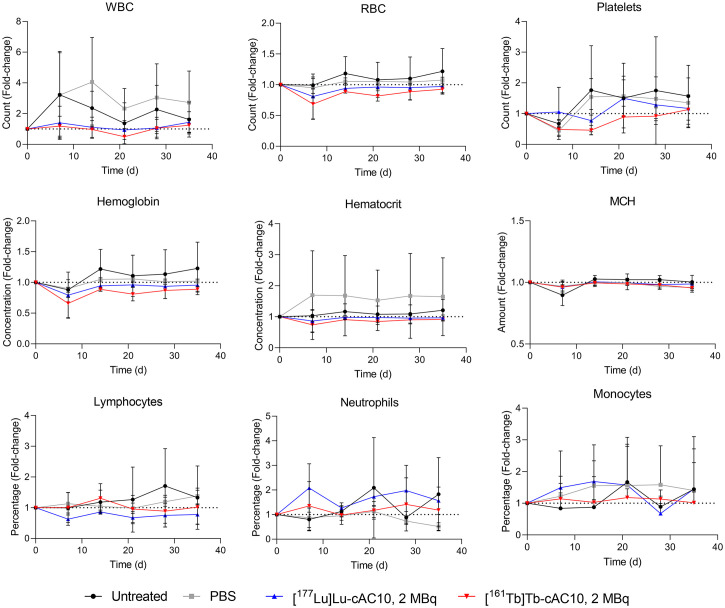
Complete blood count before treatment (day 0) and on days 7, 14, 21, 28, and 35 after intravenous injection of [^161^Tb]Tb-cAC10 or [^177^Lu]Lu-cAC10 (2 MBq, 30 μg) in non–tumor-bearing mice. Results are normalized (fold-change) to baseline values before treatment (day 0) (mean ± SD; *n* = 4). WBC = white blood cell; RBC = red blood cell; MCH = mean corpuscular hemoglobin; PBS = phosphate-buffered solution.

In terms of toxicity, conventional histomorphologic analysis of tissue sections of selected organs stained with hematoxylin and eosin revealed no obvious signs of early side effects in any of the treated groups (Supplemental Fig. 11). Animal body weight increased similarly across all groups, with no differences between control and treated mice (Supplemental Fig. 12). To further evaluate the potential acute toxicity effects derived from the radioimmunotherapy, a hematotoxicity experiment was conducted in non–tumor-bearing mice treated with conditions matching the therapeutic study. No differences in any cell blood populations or analyzed parameters were observed after treatment with either of the radioimmunoconjugates (Fig. 5; Supplemental Fig. 13; Supplemental Table 3). Moreover, these values were within the reference range for a healthy athymic nude mouse (Supplemental Fig. 13).

### Phosphoproteomic Analysis

**FIGURE 6.**
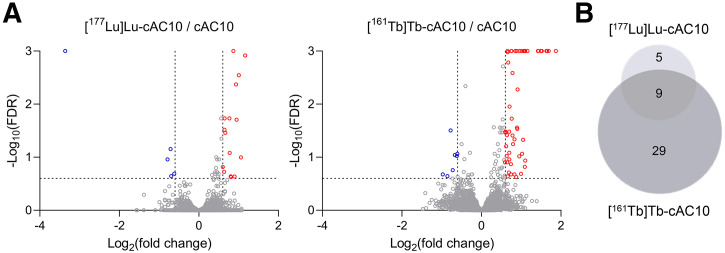
Comparative phosphoproteomic analysis of [^177^Lu]Lu-cAC10– and [^161^Tb]Tb-cAC10–treated tumors. (A) Volcano plots representing phosphopeptide abundance in response to [^177^Lu]Lu-cAC10 or [^161^Tb]Tb-cAC10 relative to cAC10 control. Red and blue dots indicate significant changes (false discovery rate ≤ 0.25; 1.5-fold change). (B) Venn diagram summarizing overlap between significantly altered phosphoproteins in response to [^177^Lu]Lu-cAC10 or [^161^Tb]Tb-cAC10 treatment.

To compare ^161^Tb- and ^177^Lu-induced signaling networks, tumors from mice treated with 2 MBq of [^177^Lu]Lu-cAC10 or [^161^Tb]Tb-cAC10 were submitted to quantitative phosphoproteomics analysis. Phosphoproteomics and proteomics analyses quantified the abundance of 12,548 phosphopeptides and 7,763 proteins. After integration of protein expression and phosphorylation profiles, the abundance of 21 and 63 unique phosphopeptides, representing 14 and 38 proteins, was significantly altered in response to [^177^Lu]Lu-cAC10 and [^161^Tb]Tb-cAC10 treatment, respectively, compared with the cAC10 control (Fig. 6A; Supplemental Tables 4 and 5). No significant changes were identified at the proteome level (Supplemental Fig. 14). Among the significantly altered phosphopeptides, 9 phosphoproteins were common to both radiolanthanides (Fig. 6B).

**FIGURE 7.**
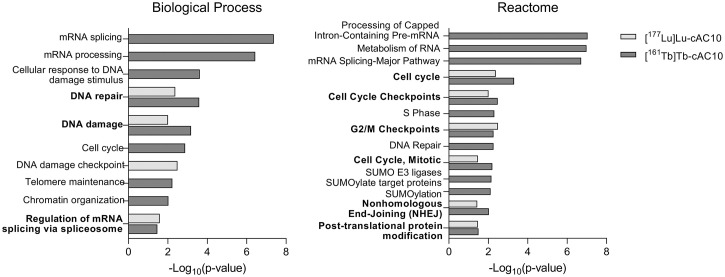
Enrichment analysis for selected relevant and common biological processes and Reactome pathway analysis of differentially altered phosphorylated proteins after [^177^Lu]Lu-cAC10 and [^161^Tb]Tb-cAC10 treatment.

Enrichment analysis revealed that both [^177^Lu]Lu-cAC10 and [^161^Tb]Tb-cAC10 induced alterations in DNA damage response/replication stress response, cell cycle arrest, and protein metabolism regulation (Fig. 7; Supplemental Tables 6–9). ^161^Tb treatment specifically altered pathways involved in SUMOylation and chromatin organization in our model. To further investigate the potential differences between both radionuclides, we directly compared the phosphopeptide abundance in [^177^Lu]Lu-cAC10– and [^161^Tb]Tb-cAC10–treated tumors, revealing significant alterations in only 18 phosphopeptides (corresponding to 12 proteins), which confirmed that the vast majority of phosphorylations were similarly affected by both radiolanthanides (Supplemental Fig. 15; Supplemental Table 10). Of these 18 phosphopeptides, 17 showed a significantly increased abundance in the ^161^Tb dataset compared with that of ^177^Lu (Supplemental Fig. 15; Supplemental Table 10).

## DISCUSSION

Radioimmunotherapy exploits the heightened radiosensitivity of lymphoma cells, which are about twice as responsive to radiation as are solid tumors (28). Unlike external beam radiotherapy, radioimmunotherapy can be used systemically in advanced stages of disease. ^161^Tb radioimmunotherapy, due to its radiodecay properties, delivers high doses of radiation to individual cells and small cell clusters, making it promising for treating hematologic malignancies (29). This radiolanthanide may also be effective in treating minimal residual disease, a small population of surviving cancer cells often present in the bone marrow and blood of patients with lymphoma after treatment, which strongly correlates with recurrence (30). The short-range electron emission of ^161^Tb could selectively eradicate these residual cells, reducing the chances of relapse.

Previous examples of the use of radioimmunotherapy in lymphoma are the aforementioned [^90^Y]Y-ibritumomab tiuxetan and ^131^I-tositumomab . Others have also explored CD30 as a target for radioimmunotherapy, mainly with the radionuclides ^90^Y and ^211^At (31). However, the radionuclides used show limited efficacy in treating disseminated diseases or minimal residual disease, whereas ^211^At’s relatively short half-life (7.2 h) makes it less suitable for use in radioimmunotherapy. The long-range β^−^-particles emitted by ^90^Y in tissue deliver insufficient energy to small tumor clusters to achieve complete remission (32). Even with ^131^I, a β^−^-emitter with a shorter range, the energy delivered to small tumor lesions remains too low (29). Moreover, the long-range β^−^-emission of ^90^Y and the significant photon emission from ^131^I increase the risk of radiation damage to healthy organs, particularly the radiosensitive bone marrow (29,33). These limitations could be overcome by the low-energy electron emission of ^161^Tb.

Our preclinical results demonstrated the potential of ^161^Tb anti-CD30 radioimmunotherapy to treat lymphomas. We successfully produced [^161^Tb]Tb-cAC10 with high yields, radiochemical purity, and affinity. In vitro studies in a panel of T-cell lymphoma cell lines revealed that ^161^Tb radioimmunoconjugate induced higher double-strand break formation, resulting in greater cytotoxicity and reduced cell survival compared with its ^177^Lu-radiolabeled counterpart. No significant differences were observed in the cellular internalization and subcellular localization of the radioimmunoconjugates across the tested cell lines, with nuclear uptake consistent with that found in previous studies (34). Recent comparisons of somatostatin receptor agonists and antagonists suggest that Auger emitters may exert greater cytotoxicity when localized at the cell membrane than when internalized, indicating nuclear localization may not be necessary to induce cytotoxicity (34). The lack of differences in the biodistribution profiles in vivo was expected due to the similar chemical properties of both radiolanthanides. Notably, high tumor accumulation of both radioimmunoconjugates was demonstrated. In terms of therapeutic effect, [^161^Tb]Tb-cAC10 had a higher antitumor effect, leading to a significant prolongation of survival, compared with equal injected activities of [^177^Lu]Lu-cAC10. This finding correlates with the 1.5-fold–higher tumor-absorbed dose calculated for [^161^Tb]Tb-cAC10, attributable to the coemission of conversion and Auger electrons. The treatment was well tolerated in this specific setting, with no signs of hematologic toxicity or bone marrow alteration. These findings are consistent with the calculated dose delivered to bone (including bone marrow), well below any toxic dose. This is particularly relevant, as bone marrow was the dose-limiting organ for both ^131^I-tositumomab and [^90^Y]Y-ibritumomab tiuxetan (35). To our knowledge, this study is the first evaluation of ^161^Tb in the treatment of any hematologic malignancy, which demonstrated its higher therapeutic efficacy compared with ^177^Lu. Future studies are needed to explore the impact of ^61^Tb in disseminated lymphoma models, where larger differences in efficacy compared with other β^−^-emitters, such as ^177^Lu, are expected.

The substantial difference in therapeutic efficacy observed between ^161^Tb and ^177^Lu prompted us to investigate the molecular effects induced in lymphoma tumors within the specific context of this study. To date, the potential differential molecular alterations that may arise from exposure to ^177^Lu and ^161^Tb have not been explored. The activation of intracellular signal transduction pathways that regulate DNA damage response and cell cycle arrest after radiotherapy plays a critical role in determining cancer response to treatment (36). To assess the signaling networks triggered by ^161^Tb and ^177^Lu in this study setting, tumors treated with 2 MBq of [^177^Lu]Lu-cAC10 or [^161^Tb]Tb-cAC10 underwent quantitative phosphoproteomics analysis. The limited phosphorylation changes reported (21 and 63 unique phosphopeptides, respectively) were expected because of the low injected activity. Importantly, 9 phosphoproteins (RBM23, ZMYND11, UTP14A, CYTIP, TP53BP1, SMC1A, MCM3, METTL16, USP28) were altered on treatment with both radiolanthanides and involved in DNA damage response (SMC1A, TP53BP1, USP28), cell cycle (MCM3, SMC1A, ZMYND11), and messenger RNA regulation (RBM23, METTL16, UTP14A). The higher number of altered phosphopeptides detected in [^161^Tb]Tb-cAC10–treated tumors correlates with the enhanced cytotoxicity, DNA damage, and therapeutic efficacy reported in this study.

Enrichment analysis of the differentially altered phosphopeptides in each group revealed that most of the induced alterations were common for both radiolanthanides (DNA damage response/replication stress response, cell cycle arrest, and protein metabolism regulation). Previous phosphoproteomic studies with different [^177^Lu]Lu-radiolabeled peptides have identified similar changes (37,38). Nevertheless, treatment with the ^161^Tb radioimmunoconjugate specifically altered SUMOylation and chromatin organization in this setting. These differences may reflect radionuclide-specific pathway activation, potentially influenced by Auger electrons inducing distinct cell responses or DNA lesions, as previously suggested (34,39). However, the differences observed between treatment with [^177^Lu]Lu-cAC10 and [^161^Tb]Tb-cAC10 in this study may have resulted from differences in deposited energy dose and subsequent signaling pathway activation, rather than radiation type. A direct comparison of phosphopeptide abundance in tumors treated with [^177^Lu]Lu-cAC10 and [^161^Tb]Tb-cAC10 confirmed that most phosphorylation events are similarly influenced by both radionuclides. Nevertheless, the vast majority of the significantly altered phosphopeptides were up-represented in the [^161^Tb]Tb-cAC10 dataset, corroborating the stronger effect of ^161^Tb radioimmunotherapy. This phosphoproteomics study demonstrated that, at the same administered activity, ^161^Tb induced more and some distinct alterations in protein phosphorylation compared with ^177^Lu.

## CONCLUSION

This work showcased the efficacy of ^161^Tb radioimmunotherapy for the treatment of CD30-positive T-cell lymphomas and represents the first study evaluating this therapeutic radionuclide for the treatment of hematologic malignancies. Overall, ^161^Tb outperformed the current benchmark ^177^Lu, likely because of the coemission of conversion and Auger electrons. Phosphoproteomics analysis comparing ^161^Tb with ^177^Lu revealed significant alterations in signaling pathways and biologic responses, confirming the superior efficacy of ^161^Tb. Our results highlight the great potential of ^161^Tb radioimmunotherapy for the treatment of CD30-positive lymphomas, including T-cell lymphomas, which have the greatest need for new therapeutic options.

## DISCLOSURE

This work was supported by the Lymphoma Challenge grant from ETH Zurich. Elisa Rioja-Blanco, Yara Banz, Christoph Schlapbach, Urban Novak, Michal Grzmil, Roger Schibli, and Martin Behe are cited as inventors in a patent application covering CD30-targeting antibody-radiopharmaceutical conjugates and their therapeutic use (EP24178074.1). Roger Schibli and Martin Behe are cofounders of Araris Biotech AG and coinventors in other patents. The mass spectrometry proteomics and phosphoproteomics data have been deposited to the ProteomeXchange Consortium via the PRIDE (https://www.ebi.ac.uk/pride) partner repository (dataset identifier PXD053937). No other potential conflict of interest relevant to this article was reported.

## Notes

KEY POINTS **QUESTION:** Can ^161^Tb be incorporated as radioimmunotherapy for effective lymphoma treatment in preclinical models? **PERTINENT FINDINGS:** [^161^Tb]Tb-cAC10 showed superior cytotoxicity and improved survival in a lymphoma mouse model compared with [^177^Lu]Lu-cAC10. Phosphoproteomic analysis revealed biologic processes and pathways altered by ^161^Tb, demonstrating its superior therapeutic effect. **IMPLICATIONS FOR PATIENT CARE:** [^161^Tb]Tb-cAC10 holds great potential for the treatment of CD30-positive lymphomas.
